# A study on antimicrobial susceptibility pattern in clinical isolates of *Staphylococcus aureus* in Eritrea

**DOI:** 10.4314/pamj.v3i1.52439

**Published:** 2009-08-17

**Authors:** Durgadas Naik, Alem Teclu

**Affiliations:** 1 Microbiologist/Associate Professor, College of Health Sciences, Asmara, Eritrea,; 2 ENT Surgeon, Sembel Hospital, Ministry of Health, Eritrea

## Abstract

**Background:**

*
Staphylococcus aureus* is a major pathogen in skin and soft tissue infections. Methicillin resistant *S.aureus* (MRSA) is prevalent in most of the countries wherever it is sought for. MRSA is one of the important pathogens implicated in hospital acquired infection. The main objectives of this study was to find out the antimicrobial susceptibility pattern of *S.aureus* isolates, the prevalence of methicillin resistant *S.aureus* (MRSA) and nasal carriage rate in healthy hospital staff.

**Method:**

A total of 278 *S.aureus* strains isolated from clinical specimens were tested for antimicrobial susceptibility and 30 anterior nares swabs from healthy hospital staff were screened for *S.aureus* organisms using standard methods.

**Results:**

High resistance was observed against ampicillin, penicillin and tetracycline. High sensitivity was recorded against amikasin, amoxicillin-c and ciprofloxacin. Of the 278 isolates 26 (9%) isolates were methicillin resistant *S.aureus* (MRSA). 17 % of the hospital staff were positive for nasal carriage of *Staphylococcus aureus* .

**Conclusion:**

Our study emphasizes the need for continuous monitoring of the antimicrobial susceptibility pattern of *S.aureus* isolates including MRSA for the selection of appropriate therapy. In Eritrea, from the present findings it appears that the spread of MRSA in community and hospital settings is limited.

## Background

*Staphylococcus aureus* is a major pathogen implicated in skin and soft tissue infections. *S.aureus* also causes abscess in deep organs, responsible for toxin mediated diseases. This organism is one of the important pathogens in hospital acquired infection. In humans, colonization of *S.aureus* is found in the anterior nares. In general, nasal carriage rate is higher in hospital staff and patients than in the community. Nasal carriage of these organisms in hospital staff provides a source for infection in hospitalized patients especially in pediatric and intensive care units. Further, the development of multiple drug resistance to this organism is posing serious threat to inpatients especially in pediatric and intensive care units in hospital [1, 2]. With the world-wide emergence of methicillin resistant *S.aureus* (MRSA), as many of the MRSA isolates are multi drug resistant, these organisms are studied with special interest. MRSA cause nosocomial infections and are associated with increased rates of illness and death [3].

Indiscriminate use of antibiotics and prolonged hospital stay are contributing factors in the emergence of multidrug resistant strains. With the emergence of MRSA and multi drug resistance, it is important to know the trend of antimicrobial resistance in any geographic area. Although, like elsewhere in the world, *S.aureus* is an important pathogen in the north-east region of Africa, the antimicrobial susceptibility pattern, prevalence of MRSA strains and carriage rate in healthy hospital staff are not known. There is no published data on these aspects. This study is an attempt to find answer to these questions.

The main objectives of this work were to study the prevalence of *S.aureus* in clinical specimens, their antimicrobial susceptibility pattern and to assess the carriage rate of *S.aureus* in healthy hospital staff.

## Method

The study was conducted in the Central Health laboratory, which serves as the National Reference Laboratory for Eritrea. Of the 278 clinical isolates, 272 isolates of *S.aureus* were from pus and 6 were from ear discharge. The anterior nares swabs were collected from 30 healthy hospital staffs who were working in the hospitals of Asmara (the capital city of Eritrea) at the time of the study.

Standard procedures were followed to isolate, identify and perform antimicrobial susceptibility pattern [4, 5]. In brief, the specimens were collected in a culturette swab. The specimens were inoculated on Blood agar and Mannitol Salt agar (Oxoid) and were incubated at 37 ^o^ C for 18–24 hours. The suspected isolated colonies were subjected to Gram’s staining. *S.aureus* organisms were confirmed mainly by positive DNAse (deoxyribonuclease) and coagulase tests. Confirmed *S.aureus* isolates were subjected to antimicrobial sensitivity testing by standard disk diffusion method as per NCCLS standards [6].

All confirmed *S.aureus* isolates were subsequently tested for methicillin resistance by using oxacillin disks (1 ug). The antimicrobials used were ampicillin (10 ug), amoxicillin-c (30 ug), ciprofloxacin (5 ug), cephalexin (30 ug), chloramphenicol (30 ug), cotrimaxozole (25 ug), gentamycin (10 ug), erythromycin (15 ug), penicillin (10 U), amikasin (30 ug), and tetracycline (30ug). American Type Culture Collection *S.aureus* 25923 was used as a reference strain. A total of 278 *S.aureus* isolates from patient specimens, and 5 isolates from 30 healthy hospital staff are included in this study.

## Results

Antimicrobial susceptibility study of *S.aureus* isolates revealed high resistance to ampicillin (85%), penicillin (77%) and tetracycline (78%). Low resistance was observed against amoxicillin-c (8%), amikasin (7%) and ciprofloxacin (5%). 32% of the isolates were resistant to chloramphenicol and gentamycin. About 23 % of the *S.aureus* isolates were resistant to erythromycin ( Table 1 ). Although our results were similar to some of the other studies [7, 8], the antimicrobial resistance to different antimicrobials varies from place to place, time to time and also depends on a number of factors like use, abuse, availability and consumption of antibiotics. In general, high resistance was observed against widely used antibiotics like penicillin. This is evident when we compared the resistance to two aminoglycoside group antibiotics gentamycin (32%) which is widely used in our setting than amikasin (7%). In one of the large studies conducted [9], 88% of the isolates were resistant to penicillin whereas only 4% were resistance to tetracycline.

In our study 26 (9%) of the *S. aureus* isolates were methicillin resistant ( Table 2 ). All the methicillin resistant S *. aureus* were also resistant to penicillin while 96% were resistant to ampicillin. Low resistance was observed to amoxicillin-c (4%), cephalexin (8%), ciprofloxacin (8%), and amikasin (15%).  None of the 5 *S.aureus* isolates from healthy hospital staff was methicillin resistant.

## Discussion

The MRSA prevalence rate shows high regional variance. This is indicated in different studies conducted in Croatia (22%), Taiwan (75– 84%), India (31–33%), Pakistan (83%), and Malaysia (40%) [9–15]. In USA there was progressive development of resistance to methicillin from 5% (1981) to 52 % (2005). In the same study, high regional variation was found from 12.5 % to 100% [16]. Similarly, in Taiwan MRSA prevalence increased from 4.3 % (1981) to 84% (2000) [15]. A high prevalence of 83% MRSA is reported from Pakistan [9]. In contrast, the prevalence rate of MRSA was found to be low in France (6%), Ireland (5%) and United Kingdom (2%) [8].

The antimicrobial susceptibility pattern of MRSA isolates also varies with place and time. In most of the studies conducted over the years, there was a clear indication of the progressive development of antimicrobial resistance to several antibiotics. In some of the studies, high resistance to ciprofloxacin was reported in MRSA isolates ranging from 46 to 99 %, [10–12] whereas in our study this was only 8 %. In Eritrea, ciprofloxacin is not widely used, and low resistance could be due to this factor. In Western Australia, high resistance was observed against erythromycin (60%) and ciprofloxacin (26%) among the MRSA isolates. The same study reported progressive development of ciprofloxacin resistance from 11% (1998) to 26 % (2002) [13]. High resistance development against erythromycin was reported (92%) in Taiwan [14]. In this study, among MRSA isolates, 27% were resistant to erythromycin and 23 % to cotrimaxozole and high resistance (88%) was observed against tetracycline. The resistance rate to co-trimaxozole varies from 0– 66% [12, 13]. In some studies resistance to cotrimaxozole was as low as 0–1% and to tetracycline 6–9% [15, 17]. Our study emphasizes the need for continuous monitoring of antimicrobial resistance development in *S.aureus* isolates including MRSA.

*S. aureus* is implicated in hospital acquired infection. Further, MRSA has emerged as a serious public health problem in all regions of the world. Community acquired MRSA infections occur as skin and soft tissue infections, whereas hospital acquired MRSA is acquired in hospitals and usually causes infections in the elderly, pediatric and immuno-compromised patients. At present, MRSA infections are treatable but there is a need to prevent the spread of MRSA in community and hospital settings. The best way to prevent the spread of *S.aureus* and MRSA in hospital settings is to screen health care takers for the presence of these organisms. The present study made an attempt to screen hospital staff for the *S. aureus* organisms. Of the 30 healthy hospital staff screened, 5 were positive for *S.aureus* and none of these was MRSA. The important reservoirs of MRSA in hospitals are infected patients and health care workers who are carriers. The carriage rate of *S.aureus* in healthy hospital staff ranges from 20–76% in other studies [10, 11, 18–20]. This study records 17 % of nasal carriage for *S.aureus* .

None of the 5 isolates of *S.aureus* obtained from hospital staff was methicillin resistant. Similar observation was made in other studies [15, 16].This is contrary to other studies where high carriage rate of 19–38% for MRSA is reported [8, 17, 18]. Some of the studies indicate that the epidemiology of MRSA is changing and hospitalization is no longer necessarily a risk factor [21, 22]. Although five isolates from 30 healthy hospital staff is very limited in numbers to draw a definite conclusion, our study also suggests this as far as MRSA is concerned.

## Conclusion

In conclusion, our study emphasizes the need for continuous monitoring of the antimicrobial susceptibility pattern of *S.aureus* isolates including MRSA for the selection of appropriate therapy. When compared to the prevalence rate of MRSA in clinical isolates in other countries, it appears that in Eritrea, the spread of MRSA in community and hospital settings is limited. However, further molecular studies are recommended to study and monitor the epidemiology of multiple drug resistant *S.aureus* and MRSA.

## Figures and Tables

**Table 1:** Antimicrobial susceptibility pattern of *S.aureus* isolates

** Antimicrobial **	*** S.aureus * (n = 278) **	
	** Number resistant **	** % resistant **
Amikasin	19	7
Amoxycillin-c	22	8
Ampicillin	236	85
Cephalexin	73	26
Chlorapmphenicol	89	32
Ciprofloxacin	15	5
Cotrimaxozole	95	34
Erythromycin	214	23
Gentamycin	89	32
Oxacillin	26	9
Penicillin	214	77
Tetracycline	216	78

**Table 2:** Resistance pattern of Methicillin Resistant *S.aureus* isolates

** Antimicrobial **	** MRSA (n = 26) **	
	** Number resistant **	** % resistant **
Amikasin	4	15
Amoxycillin-c	1	4
Ampicillin	25	96
Cephalexin	2	8
Chlorapmphenicol	7	27
Ciprofloxacin	2	8
Cotrimaxozole	6	23
Erythromycin	7	27
Gentamycin	12	46
Oxacillin	26	100
Penicillin	26	100
Tetracycline	23	88
